# Soil burial-induced degradation of cellulose films in a moisture-controlled environment

**DOI:** 10.1038/s41598-024-57436-w

**Published:** 2024-03-22

**Authors:** Shaida S. Rumi, Sumedha Liyanage, Noureddine Abidi

**Affiliations:** Department of Plant and Soil Science, Fiber and Biopolymer Research Institute, Texas Tech University, Lubbock, TX 79409 USA

**Keywords:** Biodegradable films, Cellulose, Bioplastics, Compost, Soil burial, Waste cotton, Environmental impact, Environmental sciences, Materials science

## Abstract

In this study, the biodegradability of cellulose films was evaluated in controlled-moisture soil environments. The films were prepared from low-quality cotton fibers through dissolution in DMAc/LiCl, casting, regeneration, glycerol plasticization, and hot-pressing. Two soil burial degradation experiments were conducted in August 2020 (11th August to 13th October) and March 2021 (24th March to 24th July) under controlled moisture conditions to assess the biodegradation behavior of cellulose films. The films were retrieved from soil beds at seven-day intervals, and morphological and physicochemical changes in the films were investigated. The results indicated that the cellulose films exhibited gradual changes starting on Day 7 and major changes after Day 35. Stereomicroscopy images showed the growth and development of fungal mycelia on the surface of the films, and FTIR spectroscopy confirmed the presence of biomolecules originating from microorganisms. The tensile strength and elongation of cellulose films were significantly reduced by 64% and 96% in the first experiment and by 40% and 94% in the second experiment, respectively, during the degradation period. Degradation also significantly impacted the thermal stability (14% and 16.5% reduction, respectively, in the first and second studies) of the films. The cellulose-based films completely degraded within 63 days in late summer and 112 days in spring. This study demonstrates that, unlike synthetic plastics, films prepared from low-quality cotton fibers can easily degrade in the natural environment.

## Introduction

The inevitable environmental and health issues associated with the excessive use of synthetic plastic in everyday life have become a global problem. With large-scale production and accessibility at a meager cost, especially with the worldwide shift from reusable containers to one-time-use items, it has become a rapidly growing problem in municipal solid wastes^1^. In addition, significant amounts of plastic materials are disposed of in the environment^2^, and their recovery for reuse or reprocessing remains minimal^3^. Since the constituent monomers of commonly used plastics are derived from fossil hydrocarbons, they resist microbial degradation^4,5^ and persist in the natural environment or landfills for hundreds of years^6^. Their extensive presence in nature has a broad spectrum of unintended consequences^3,7^. Although waste management through incineration, combustion, or pyrolysis can be used to permanently eliminate plastic waste, it is associated with many negative impacts, including the release of toxic compounds and greenhouse gases into the atmosphere^1,2,8^.

The growing interest in replacing synthetic plastic with sustainable alternatives to alleviate environmental problems has propelled researchers to develop bioplastics that are easily decomposed or recycled. Bioplastics can be degraded by living microorganisms, such as bacteria, fungi, and yeast^9^. They are considered one of the most promising solutions to reduce plastic litter. Microorganisms feed on biodegradable plastics as their carbon source, followed by the subsequent assimilation or mineralization of the plastics, causing the ultimate disappearance of the material from the natural environment^10^. However, the use of bioplastic is limited owing to its high production cost, which can be manageable if low-cost raw materials are used as feedstock^11^. Biomass is a common raw material for producing bioplastics^12^. Cellulose, a fascinating biopolymer with exceptional properties, is extracted from lignocellulosic biomass^13^. Consequently, cellulose is an inexhaustible raw material for the production of eco-friendly bioplastics^13^.

Cotton and cotton byproducts are attractive sources of almost pure cellulose. In a previous study, we sought to develop an eco-friendly alternative to petroleum-based plastic films using low-quality cotton fibers (low maturity and low strength), which are not suitable for traditional textile use^14^. We optimized the production parameters and fine-tuned the physicochemical properties of the resultant films. Our study emphasized the production of cellulose films to create a niche market for low-quality cotton fibers and bring a high profit to the cotton industry.

The biodegradation behavior of bioplastics is affected by their structural complexity and composition and the environment in which the bioplastic is disposed of. Environmental factors, such as temperature, moisture, and pH, significantly impact the biodegradation of bioplastics^8^. In this study, we focused on understanding the degradation behavior of cellulose films disposed of in a moisture-controlled environment. Since plastic is widely discarded in terrestrial environments, we investigated the biodegradability of cellulose films in soil burial conditions. We primarily analyzed the gradual changes in the morphological, biochemical, thermal, and mechanical properties of cellulose films during the degradation process. The aim of the present study was to demonstrate that the dissolution of cotton fibers and the regeneration of cellulose can lead to films that can undergo biodegradation in the soil in a reasonable time. This opens new applications of these films as agricultural mulches or soil cover plastics for growing vegetables and fruits. At the end of the growing season, cellulose-based plastic films can remain in the soil and undergo biodegradation with no residues.

## Results and discussion

### Experimental setup

**Figure 1:**
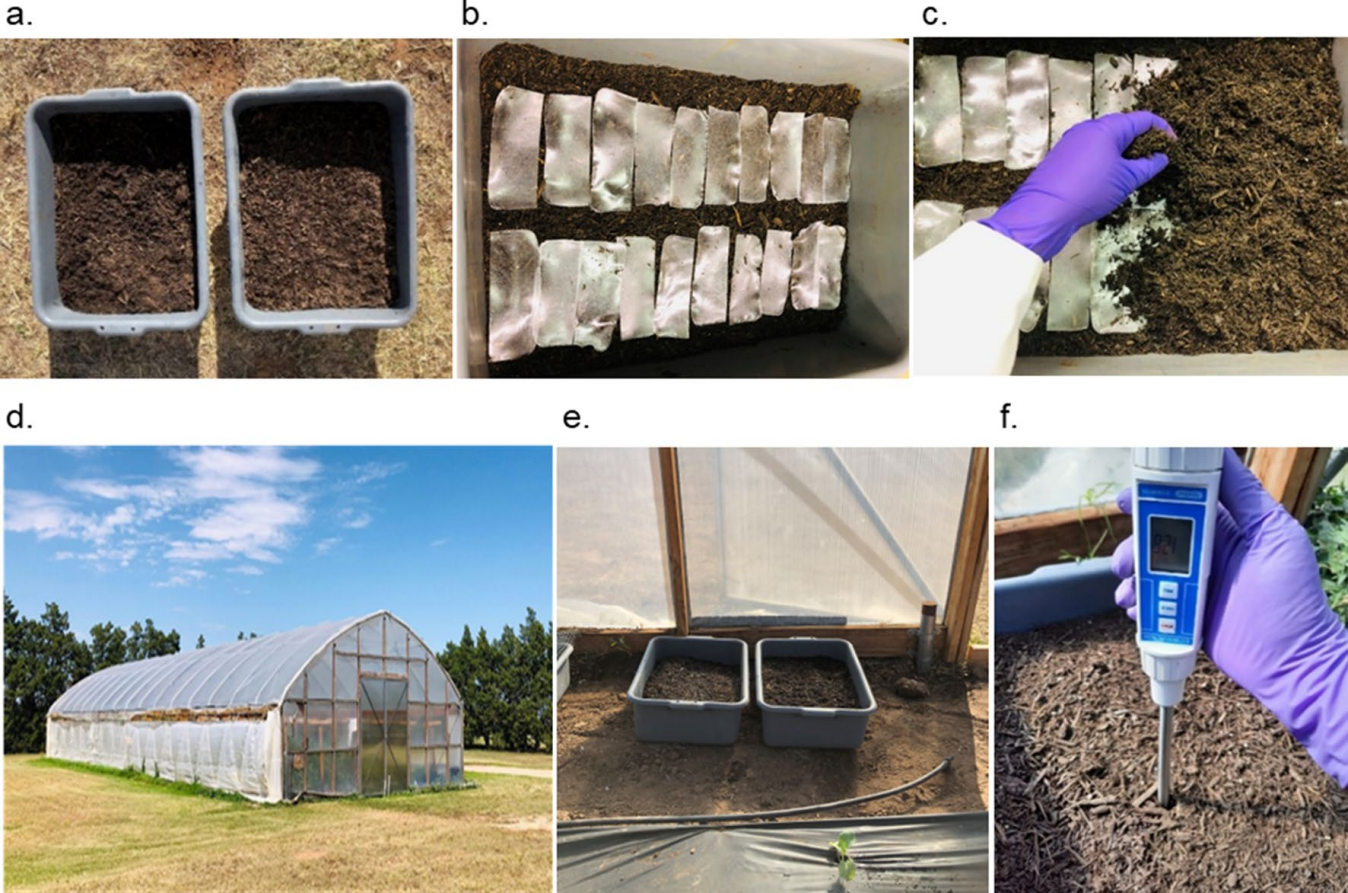
Degradation study of cellulose films under a controlled moisture environment. (**a**) preconditioned soil trays (12 ± 2% moisture), (**b**) cellulose films placed on the moist soil bed 5 cm below the soil surface, (**c**) soil burial of cellulose films, (**d**) high tunnel, (**e**) sample trays placed inside the high tunnel, and (**f**) soil moisture measurement using the digital soil moisture meter.

Soil burial experiments were conducted in late summer and spring to study the effect of environmental conditions on the soil burial-induced degradation of cellulose films. The first experiment was conducted in 2020 from 11th August to 13th October, and the second was conducted in 2021 from 24th March to 14th July. Figure 1 shows the experimental setup of the study. During the conditioning period, the soil beds were watered as needed and mixed several times to ensure uniform distribution of moisture in each soil bed. Soil mixing helped prevent moisture buildup in localized areas of soil beds. Soil moisture readings were regularly collected from several locations of each soil bed, and it was ensured that each moisture meter reading was 12 ± 2%. After the soil beds were conditioned for 7 days, the cellulose films were buried inside the soil bed 5 cm below the soil surface. Then, the experiment was conducted inside a high tunnel to prevent excessive moisture buildup in the beds during rainfall. Watering was continued with a watering can (sprinkling can), which was equipped with a long spout and a sprinkler head. It helped break up water stream into droplets to avoid much water pressure on the soil. It also helped uniform distribution of water in the soil beds, preventing moisture buildup in localized areas. It required adding approximately 500 ml of water every two days to maintain 12 ± 2% moisture. The cellulose films were retrieved at 7-day intervals in the first experiment and up to 56 days in the second experiment. Since the degradation period was longer than expected in the second experiment, the sample retrieval interval was increased to 14 days after Day 56 until the end of degradation.

### Changes in sample weight

The effect of soil burial on sample weight was investigated using the weights recorded for each film before the experiment and after they were retrieved from soil beds. In the first experiment, the recorded weight losses of samples retrieved on Days 7, 14, 21, 28, 35, 42, 49, and 56 were approximately 47.5, 57, 66, 68, 74, 78, 82, and 87%, respectively. In the second experiment, the recorded weight losses of samples retrieved on Days 7, 14, 21, 28, 35, 42, 49, 56, 70, 84, and 98 were 72, 74, 75, 76, 76, 78, 79, 83, 84, 86, and 90%, respectively. In both experiments, the initial weight loss during the first week of soil burial was significantly higher than those in the consecutive retrievals. This initial reduction in sample weight was primarily attributed to the removal of glycerol, which FTIR and TGA analysis of the retrieved samples could further verify. Since glycerol is highly miscible with water, regular watering and soil moisture resulted in the rapid removal of glycerol from the films. The further gradual change in sample weight was related to the removal of the remaining glycerol and microbial assimilation of cellulose as a carbon source, especially at the later stages of the soil burial experiments.

### Morphological changes in cellulose films

**Figure 2:**
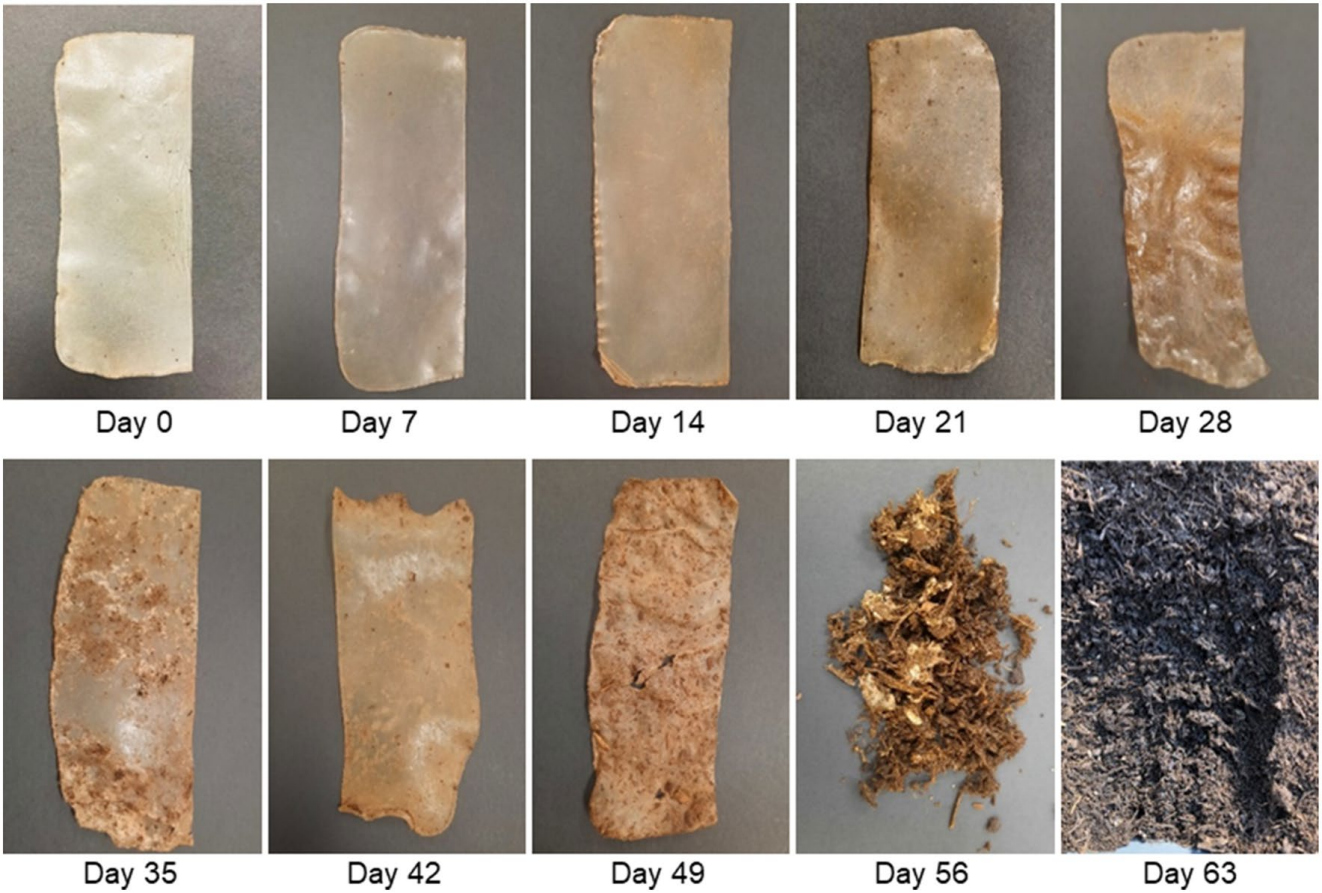
Visual changes of soil-buried cellulose films (control film (day 0) and films retrieved on days 7, 14, 21, 28, 35, 42, 49, and 56).

Figure 2 shows the gradual changes that occurred in the sample appearance during the first soil burial experiment. The samples retrieved up to Day 14 did not show a significant change in appearance except for minor discoloration. Sample discoloration continued to increase with retrieval time, possibly due to the adsorption of soil particulates or microbial attack. The adsorption of soil particulates can cause yellowing/discoloration, especially in the presence of soil moisture^15^. The discoloration of the films also indicates a change in the morphology of films due to microbial attack^6,16^. The presence of microorganisms causes pH alterations in materials due to the hydrolytic actions of microbial enzymes, which manifests as discoloration^17^ and later leads to material degradation.

Furthermore, considerable sample shrinkage was observed, possibly due to the removal of glycerol in the moist soil environment, leading to increased sample stiffness. From Day 35 onwards, some whitish spots/areas appeared on samples that could be assumed to be fungal mycelia, indicating that the cellulose films support the growth of microbes. The white regions spread over the films retrieved in consecutive weeks. In addition, the samples looked increasingly wrinkled, thin, and weak, with noticeable holes appearing on the films retrieved on Day 49. On Day 56, the most significant deterioration could be noticed, and samples could not be recovered as a single piece. On Day 63, not even small pieces of cellulose films could be found in soil beds, confirming the complete assimilation of films by microorganisms.

Similar to the first experiment, the samples retrieved during the second experiment showed only slight discoloration within the first few weeks. The sample discoloration was much more pronounced after Day 35 (supplementary materials-Fig. S1). White and black microbial colonies were also noticed on Day 28, and they gradually spread on the cellulose films with increasing burial time, suggesting the proliferation of microorganisms. As a result, the films became lighter and fragile over time. However, these changes occurred more slowly in the second experiment than in the first experiment, and the films took 112 days to complete the degradation process.

**Figure 3:**
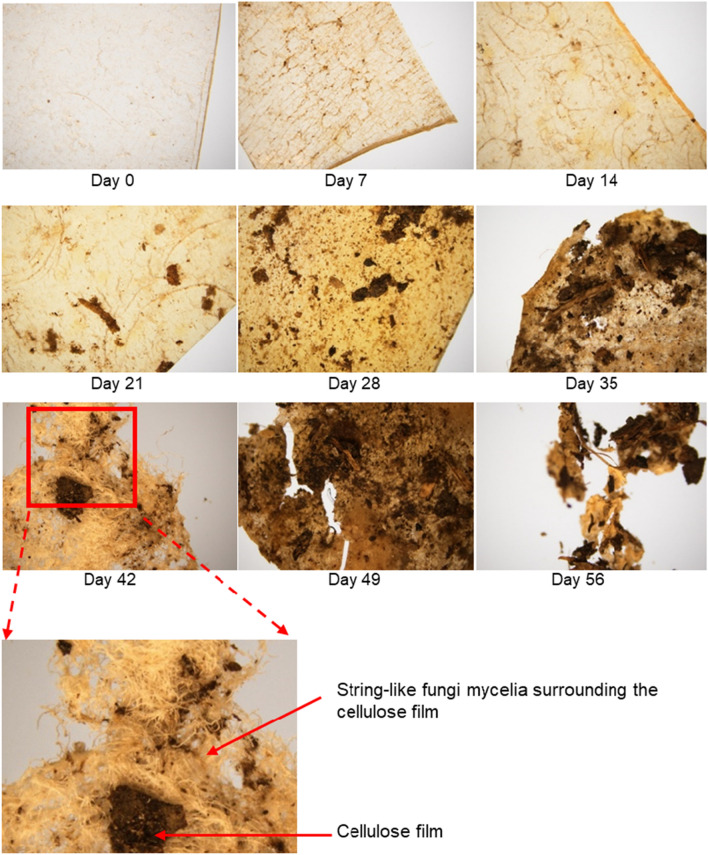
Stereo microscopy images of cellulose films retrieved during soil burial experiments.

Figure 3 shows the stereomicroscopy images of cellulose films retrieved during the first soil burial experiment. Adherence of soil particles and sample discoloration are clearly visible in these images. In addition, the presence of fungal mycelia and holes/cracks were noticeable from Day 35 onwards. In particular, the samples retrieved on Day 42 showed the presence of highly branched whitish structures surrounding the cellulose films (Fig. 3b), which appeared similar to SEM micrographs of fungal mycelia reported in a previous study^18^. Fungal mycelia are the radiating structures of hyphae, which usually spread over a substrate for nutrient retrieval and thereafter penetrate the substrate via the hyphal tips with the help of the high turgor pressure developed by the rigid hyphal wall^19^. Highly branched mycelia indicate a high surface area, and it is assumed that the productivity of degradation enzymes increases via highly branched mycelia, as they can effectively interact with a larger area of the substrate^18^. The stereomicroscopy images suggest that cellulose films support microbial growth, leading to gradual degradation and subsequent assimilation of cellulose films within 63 days.

The samples retrieved during the second experiment showed similar discoloration, deterioration, fragmentation, and degradation trends. However, the complete degradation of the cellulose films took a long time compared to the first experiment (supplementary materials-Fig. S2). The growth of fungal mycelia was observed from Day 28 onwards. Although no holes or cracks were observed until Day 98, the colonization and proliferation of fungi were better observed in the second experiment.

According to SEM analysis of the cellulose films, the smooth and uniform film surface started to change as early as Day 7 (supplementary materials-Fig. S3). The shiny particles that appeared on some of these micrographs were sand particles that adhered to the sample surface when the samples were buried in the moist beds. The surface became uneven, irregular, and rougher with burial time. Similar to the stereomicroscopy images, an intense change in surface morphology was noticed from Day 35 onwards. A highly disintegrated surface with deep noticeable cracks was revealed on Day 56, causing the sample to breakdown. A somewhat similar change in the surface morphology of a soil-buried PLA/acetyl tributyl citrate/chitosan composite was reported in a different study^20^.

**Figure 4:**
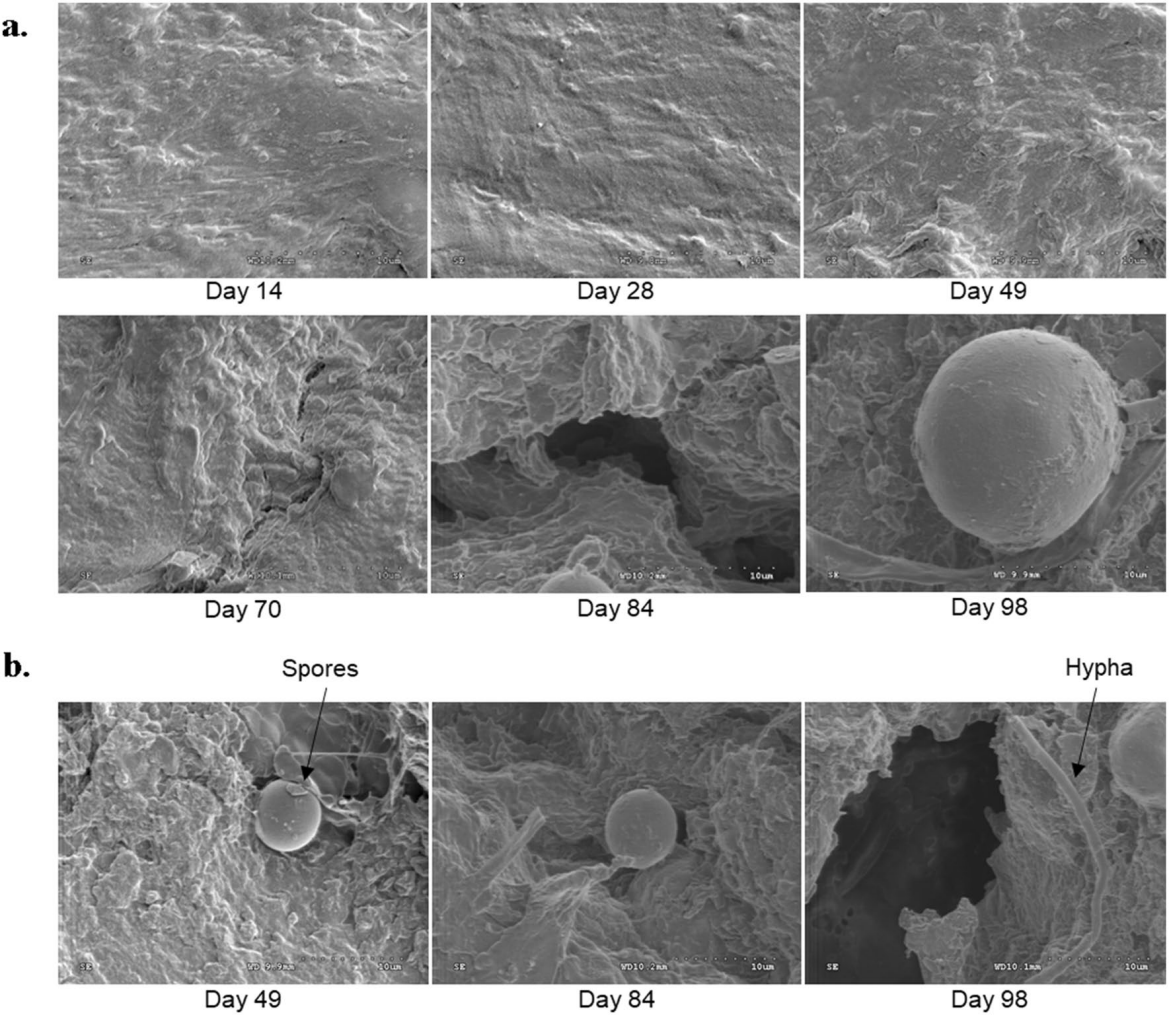
Field emission scanning electron microscopy (FESEM) analysis of retrieved cellulose films. FESEM micrographs of retrieved cellulose films at days (**a**) 14, 28, 49, 70, 84, and 98 at higher magnification (5K) and (**b**) 49, 84, and 98 at lower magnification (3.5K).

Figure 4 shows the FESEM micrographs of cellulose films retrieved on selected days during the second soil burial experiment. At 5K magnification, the changes in surface morphology and growth of fungal mycelia were clearly visualized (Fig. 4a). The images also showed that microbial attack started from the material surface, gradually disrupting the substrate's inner structure. Similar to the first experiment, the changes in cellulose films intensified with burial time. Smooth and round spores were observed starting from Day 49, which could be ascospores or conidia produced during the reproduction of fungi^21^. More information was obtained from images collected at 3.5K magnification (Fig. 4b), showing that the filamentous structures that appeared next to round structures were hyphae^22^. These branched structures make up the fungal mycelia.

The observations described above revealed the biodegradation process of the soil-buried cellulose films through visible changes, such as discoloration, surface roughness, colonization, and proliferation of microbes on the substrate, substrate fragmentation, and finally, assimilation. Thus, all the morphological observations demonstrated three primary stages of biodegradation^23^. In the first stage of biodegradation, microorganisms started growing around the surface of the substrate. Therefore, the surface of the cellulose films deteriorated first. The substrate-specific catalytic activity of microorganisms turned the complex polymer structure into low molecular weight oligomers/monomers in the second stage, leading to the fragmentation of the cellulose films noticeable via visual observations, stereomicroscopy, and SEM images. In the third stage, microorganisms assimilated simple molecules to obtain energy and converted cellulose into CO_2_, water, and biomass. As a result, the cellulose films were completely degraded within 63 and 112 days during the first and second experiments, respectively.

Compared to the first experiment conducted in August 2020, the degradation process took a long time in the second experiment conducted in March 2021, possibly due to the differences in microbial activity in late summer/early fall and spring weather conditions. In particular, the temperature directly affects physiological activities of soil microorganisms^24^ and their accessibility to food sources (substrate). It has been reported that an upward shift in temperature increases the soil microbial population, while a downward shift in temperature decreases the microbial population^25^. Therefore, seasonal changes in temperature possibly affected the activity of the soil microbial population. Production of the cellulase enzyme required for cellulose degradation is also influenced by the temperature^26,27^. It has been reported that cellulolytic microorganisms have a better potential to produce cellulase enzymes between 25 and 45 °C^26–29^.

**Figure 5:**
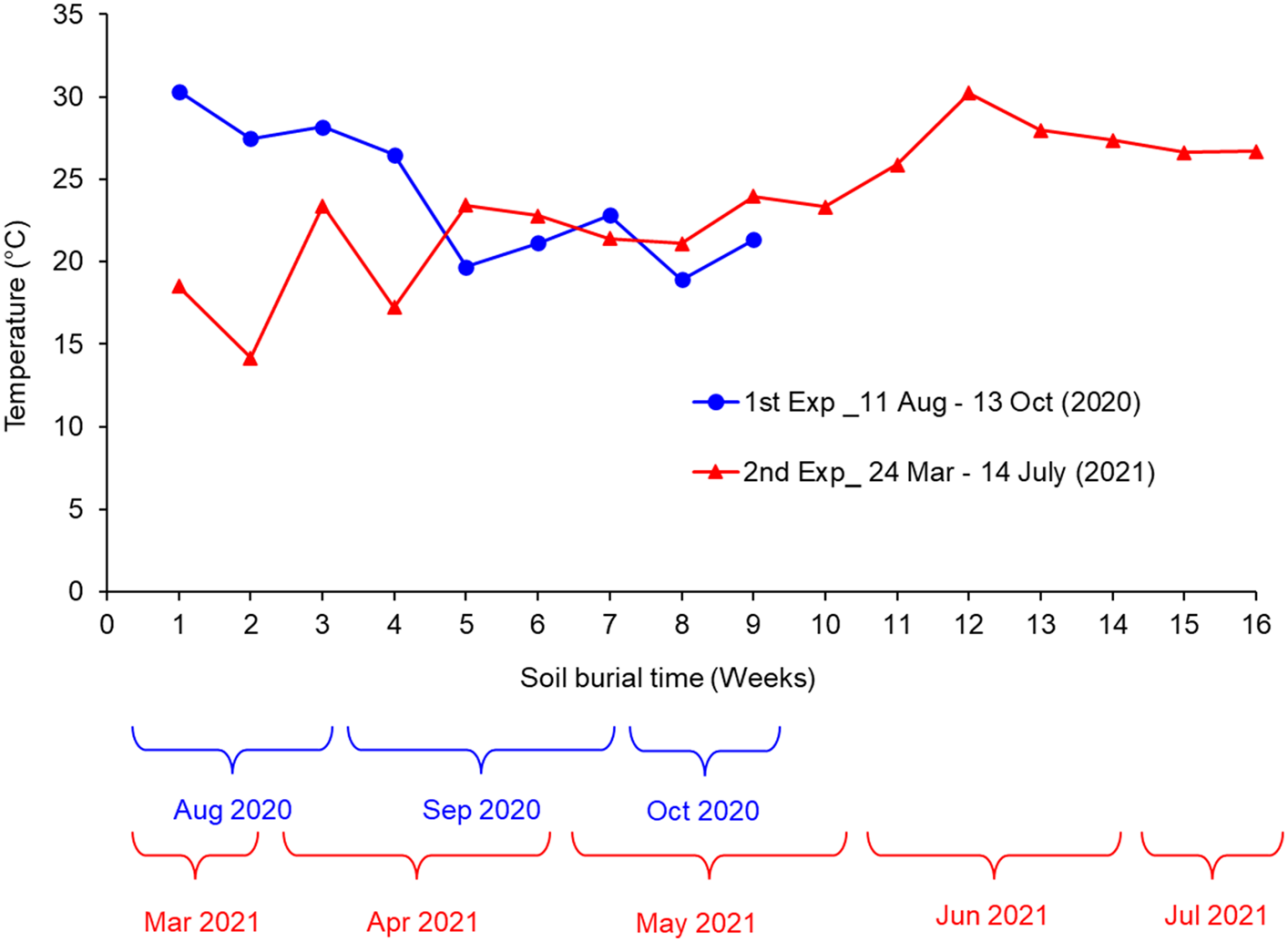
Weekly temperature recorded during the first and second soil burial experiments.

Figure 5 shows the weekly average temperatures recorded during the first and second soil burial experiments. There was a downward shift in the temperature during the first experiment, and an opposite trend was noticed in the second experiment in the spring 2021. In the first experiment, the initial four weeks were warmer, and the temperature stayed within the range ideal for cellulase enzyme production, triggering the fast degradation of cellulose films. In contrast, the average temperature remained below the optimal range through the first eight weeks of the second experiment. Moreover, the weekly, daily, and day and night temperature fluctuations were intense during the first four weeks of the second experiment. Temperature fluctuations hinder microbial activities and are also deleterious to their viability^25^. Consequently, a longer time was required to degrade the cellulose films during the second soil burial experiment.

### FTIR spectroscopy characterization

FTIR spectroscopy is highly sensitive to biochemical profiles and to changes in substrates. In this study, FTIR spectroscopy demonstrated major changes associated with the removal of glycerol, adherence of soil organic and mineral particles, and microbial growth on the cellulose substrates.

**Figure 6:**
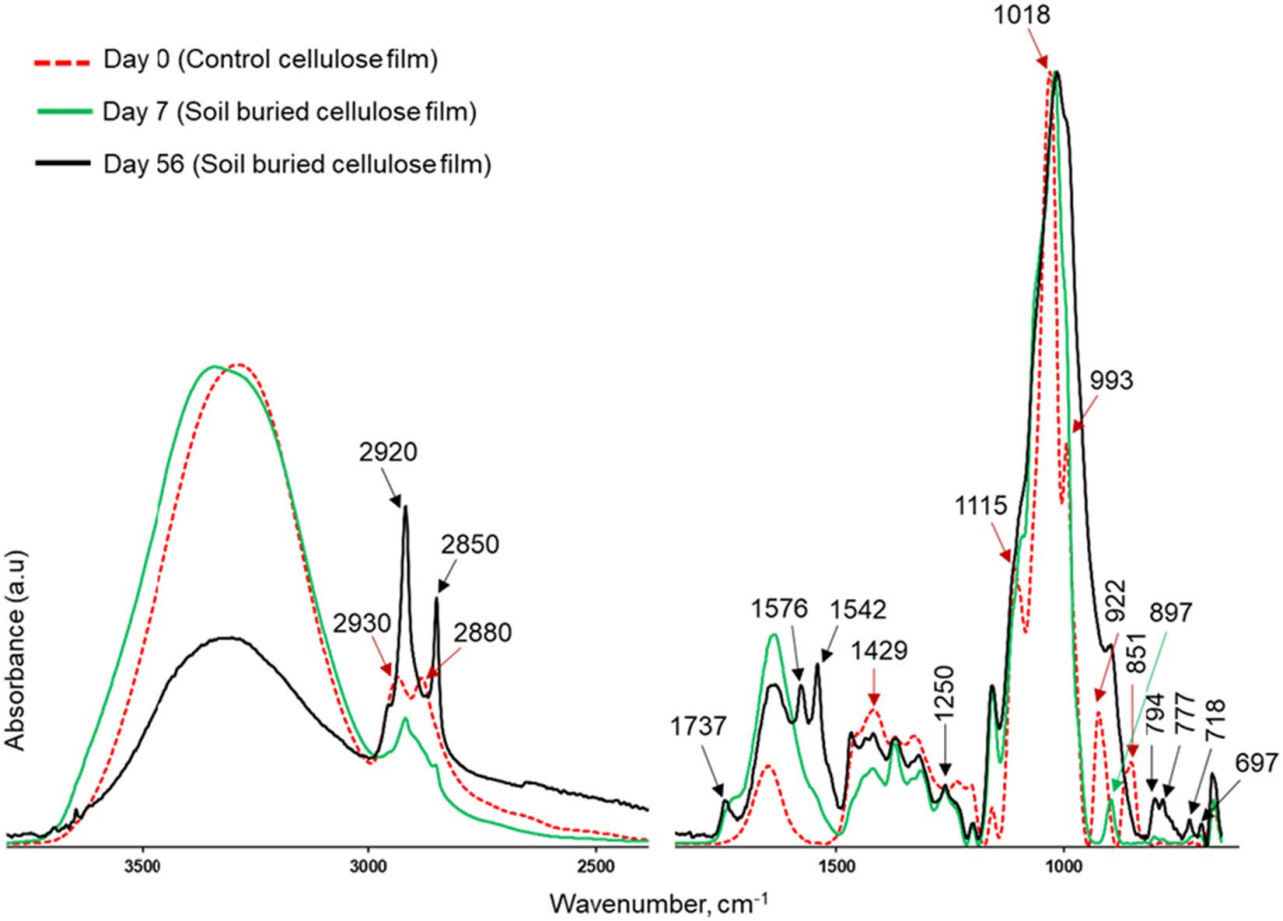
FTIR spectra acquired from control cellulose film (day-0) and the films retrieved at days 7 and 56 representing early and late degradation stages, respectively.

Figure 6 shows FTIR spectra collected from the control film and films retrieved at Days 7 and 56, representing early and late degradation stages, respectively. The control cellulose film showed major IR bands at 2930, 2880, 1115, 993, 922, and 851 cm^−1^ originating from glycerol. These vibrations quickly disappeared during the initial stages of the soil burial experiment, as glycerol is highly miscible with water and is consequently removed with soil moisture. The infrared band at 897 cm^−1^ is assigned to the β-linkage of cellulose, and it remained masked by the infrared band at 922 cm^−1^ (originating from glycerol) in the infrared spectra of the control film. The infrared band at 897 cm^−1^ became visible in the infrared spectrum of the film retrieved on Day 7, suggesting the removal of glycerol.

It was also apparent that new infrared bands appeared in the spectra, particularly in samples retrieved in the later stages of soil burial-induced degradation. The vibrations noticed in the spectral region between 815 and 690 cm^−1^ were attributed to organic and mineral particles (e.g., ~ 692 cm^−1^: quartz^30^; those at ~ 680 cm^−1^ were vibrations of Si–O–B from borosilicate^31^; those at ~ 800 cm^−1^ were quartz^32^; and those at ~ 711 cm^−1^ were calcite^32^). These spectroscopic features agree with visual observations and stereomicroscopy images, in which the adhesion of soil particles was clearly visible.

FTIR spectra of the retrieved cellulose films also showed new infrared absorption bands, most prominently in the spectral region between 3000 and 1500 cm^−1^. Obvious changes could be observed in the regions between 3000–2800, 1800–1485, and 1485–1185 cm^−1^. The intense infrared bands at 2920 and 2850 cm^−1^, assigned to CH_2_ asymmetric and CH_2_ symmetric stretching, provided information about the presence of lipids in the cellulose films^33–35^. The infrared band at 1737 cm^−1^ corresponds to the carbonyl band (C=O) of fatty acid esters^34^. The infrared bands at 1576 and 1542 cm^−1^ are assigned to the amide II bands (N–H and C–N groups) of proteins^34^. The spectral changes in the region 1485–1185 cm^−1^ suggested the presence of protein, lipid, or phosphate compounds^33^. In particular, the P=O vibration band at 1250 cm^−1^ indicated the presence of nucleic acids in the retrieved cellulose films^33–35^. From these spectroscopic changes related to lipids, proteins, polysaccharides, phospholipids, and nucleic acids (at 2920, 2850, 1737, 1576, 1542, and 1250 cm^−1^), it was assumed that the cellulose films served as substrates for microbial growth. It is plausible that microbes fed on the cellulose substrates and promoted their degradation process. As discussed earlier, the presence of microorganisms or fungi can be verified from stereomicroscopic observation. However, the intensities of the aforementioned infrared bands did not follow a clear trend with burial time, possibly due to the nonhomogeneous and uncontrolled distribution of microorganisms in the soil beds.

The intensity of the absorption band at 1429 cm^−1^, referred to as a crystalline absorption band of cellulose^14^, was relatively lower in the retrieved cellulose films than in the control cellulose film. This result suggested the disruption of the crystalline structure of cellulose during burial. Moreover, the absorption band at 897 cm^−1^, which was prominent at the initial stage of degradation, was broadened and existed as a shoulder with increasing soil burial time, implying the highly disordered structure of cellulose^36^. Similar to the first experiment, FTIR spectroscopic analysis of samples from the second experiment showed major spectroscopic changes due to the removal of glycerol, microbial growth, and accumulation of soil mineral particles (supplementary materials**- **Fig. S4). However, the intensities of the vibration bands originating from soil microorganisms were lower than those in the first experiment. As discussed earlier, this could be explained by the activity of soil microorganisms, which was likely higher in the first experiment than in the second experiment due to temperature differences.

### Tensile testing of cellulose films

**Figure 7:**
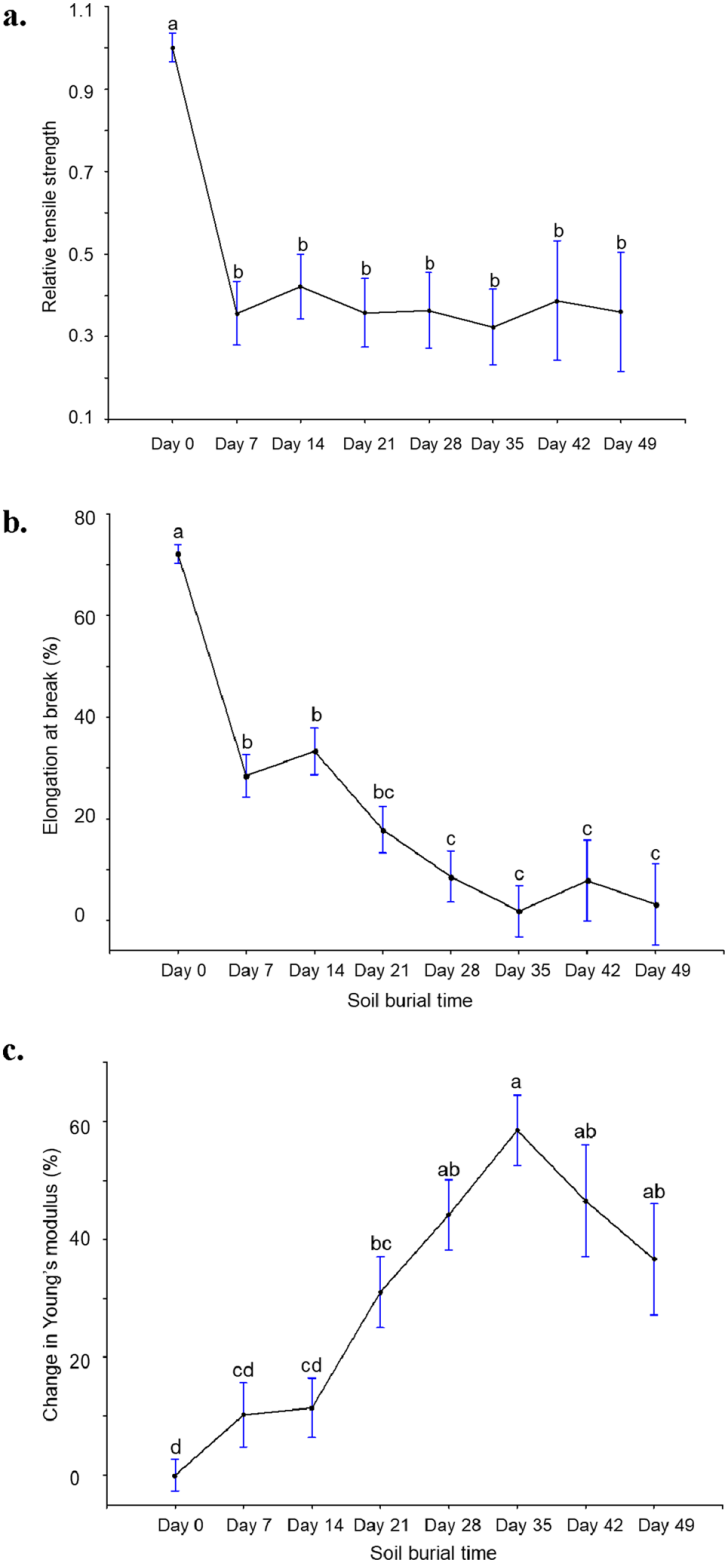
The changes in tensile properties of cellulose films retrieved during the soil burial degradation study. (**a**) Relative tensile strength, (**b**) elongation at break (%), and (**c**) change in Young’s modulus. Values not followed by the same letter significantly differ at α = 0.05.

The tensile properties of the control cellulose films were comprehensively discussed in our previous study^14^. Figure 7 shows the effect of burial time on changes in the tensile strength, strain, and stiffness of cellulose films. There was a drastic reduction in the relative tensile strength within 7 days of soil burial, accounting for approximately 65% less than that of the control films (Fig. 7a). No significant change in the relative tensile strength was observed thereafter. Shogren et al.^16^ reported a similar reduction in the tensile strength of soil-buried starch/PLA films. The authors observed a sharp initial drop in strength, which did not change much for the rest of the burial time. Lv et al.^37^ also reported an abrupt reduction in the tensile strength of PLA/starch/wood flour films during the initial stage of soil burial-induced degradation. Moreover, the elongation at break showed a 60% loss within 7 days and a  ~ 97% loss within 35 days (Fig. 7b). In an experiment conducted with PLA films, Rudnik et al.^38^ reported a 72% decrease in elongation at break within 30 days of soil burial. The loss of elongation at break was associated with the increasing Young’s modulus of the samples. In fact, the loss of glycerol during the soil burial process reduced the flexibility of the cellulose samples and made them stiff. As a result, all the samples buried in the moist soil beds presented a relatively higher Young’s modulus than the control (Fig. 7c). A similar result was reported by Vasile et al.^20^ for tributyl o-acetyl citrate plasticized soil buried PLA films. An increase in Young’s modulus was noticed with an increase in burial time until Day 35, and it did not change significantly after that.

Overall, the changes in tensile strength, elongation, and Young’s modulus were mainly associated with the loss of plasticity of the cellulose films or the removal of glycerol and microbial degradation of cellulose molecules. The hydrophilic nature of cellulose and the presence of glycerol in the films made them more susceptible to the growth of microorganisms^39^. Initial water absorption from the moistened soil during the experiment weakened the material by increasing the free volume between the cellulosic chains^6,16^. In addition, water served as a medium for microorganisms to enter the polymer network, facilitating the colonization of microbes and resulting in a deterioration in mechanical properties^6,39^. With an increase in soil burial time, microorganisms opened the structure of cellulose^15^, which resulted in macroscopic degradation and a loss of tensile properties. Slight fluctuations in tensile data were noticed, suggesting variability in the microenvironment in soil beds, which may impact microbial activities. From Day 49 onwards, cellulose films started losing their integrity due to microbial assimilation. Therefore, tensile properties were not monitored beyond this point in time.

Tensile analysis of the second soil burial experiment also showed similar changes in elongation at break (%) and Young’s modulus (supplementary materials- Fig. S5b,c). However, the relative tensile strength of cellulose films showed significantly higher values than the control film (supplementary materials- Fig. S5a), possibly due to the removal of glycerol, which made the films stiffer. After Day 14, the strength of the cellulose films significantly decreased. The FTIR spectroscopy data verified the presence of microorganisms from Day 7 onwards. However, due to the presence of lower temperature conditions, microbial activity was not intense enough to reduce the mechanical strength of the films until Day 14.

### Thermogravimetric analysis of cellulose films

**Figure 8:**
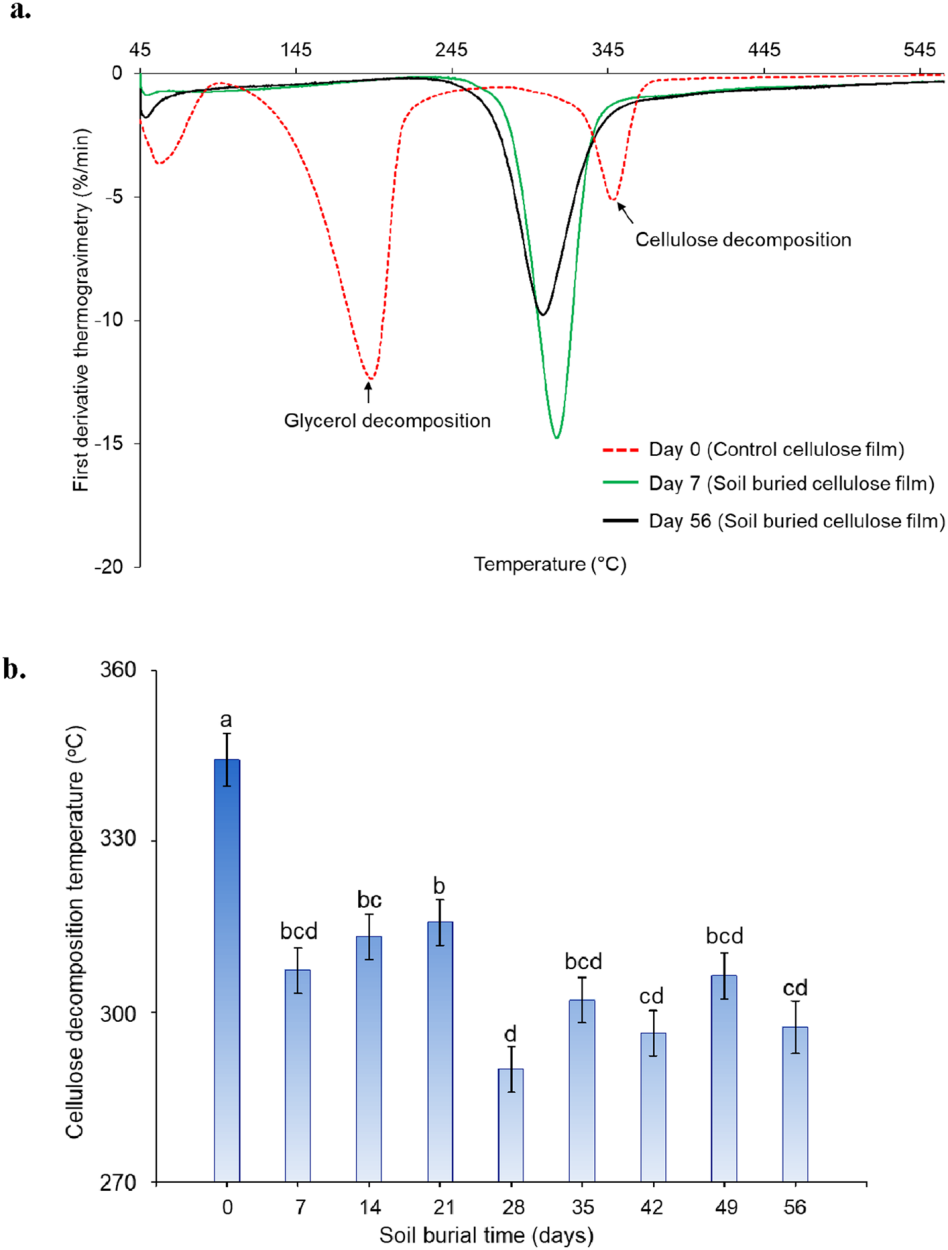
Thermogravimetric analysis (TGA) of the cellulose films. (**a**) First derivative thermogravimetry of the control and the soil buried cellulose films (retrieved at days 7 and 56) and (**b**) changes in cellulose decomposition temperatures (DTG-Peak) of the retrieved soil buried cellulose films. Values not followed by the same letter are significantly different at α = 0.05.

Figure 8a shows representative thermograms of the control and retrieved cellulose films. The thermograms of the control cellulose films showed two main decomposition regions at approximately 200 and 340 °C that were attributed to glycerol and cellulose decomposition, respectively^14^. However, the thermograms of the retrieved cellulose films did not show a weight loss region attributed to glycerol decomposition, confirming the removal of glycerol, as discussed earlier. According to the TGA data, soil burial-induced degradation significantly reduced the thermal stability of the cellulose films). The thermal decomposition (DTG-Peak) of all the soil-buried cellulose films was lower than that of the control cellulose film (Fig. 8b). The onset thermal decomposition (T_onset_) of the samples also exhibited a similar trend to that of the peak decomposition temperature (supplementary materials—Fig. S6). However, the cellulose decomposition temperature did not follow a specific trend with increasing burial time. The slight variations in cellulose decomposition temperature with increasing soil burial time could be associated with two main factors. First, the microbial population in the soil bed (or around cellulose films) is not homogeneous, and therefore, the microbial assimilation of each cellulose film is not homogeneous. Second, the removal of attached soil particles from the retrieved cellulose films became increasingly difficult with degradation time. The presence of these foreign particles might have impacted the cellulose decomposition temperature. Similar to the first experiment, the decomposition (DTG-Peak) of samples in the second experiment occurred at lower temperatures for all the retrieved samples compared to that of the control films (supplementary materials—Fig. S7), and the thermal stability of the soil-buried cellulose films was significantly reduced starting from Day 7 in the second experiment. As we discussed earlier, the slight variations in the cellulose decomposition temperatures could be associated with heterogeneous microbial distribution in soil beds (which may result in uneven degradation of cellulose films) and the presence of soil particles adhering to the cellulose films.

**Figure 9:**
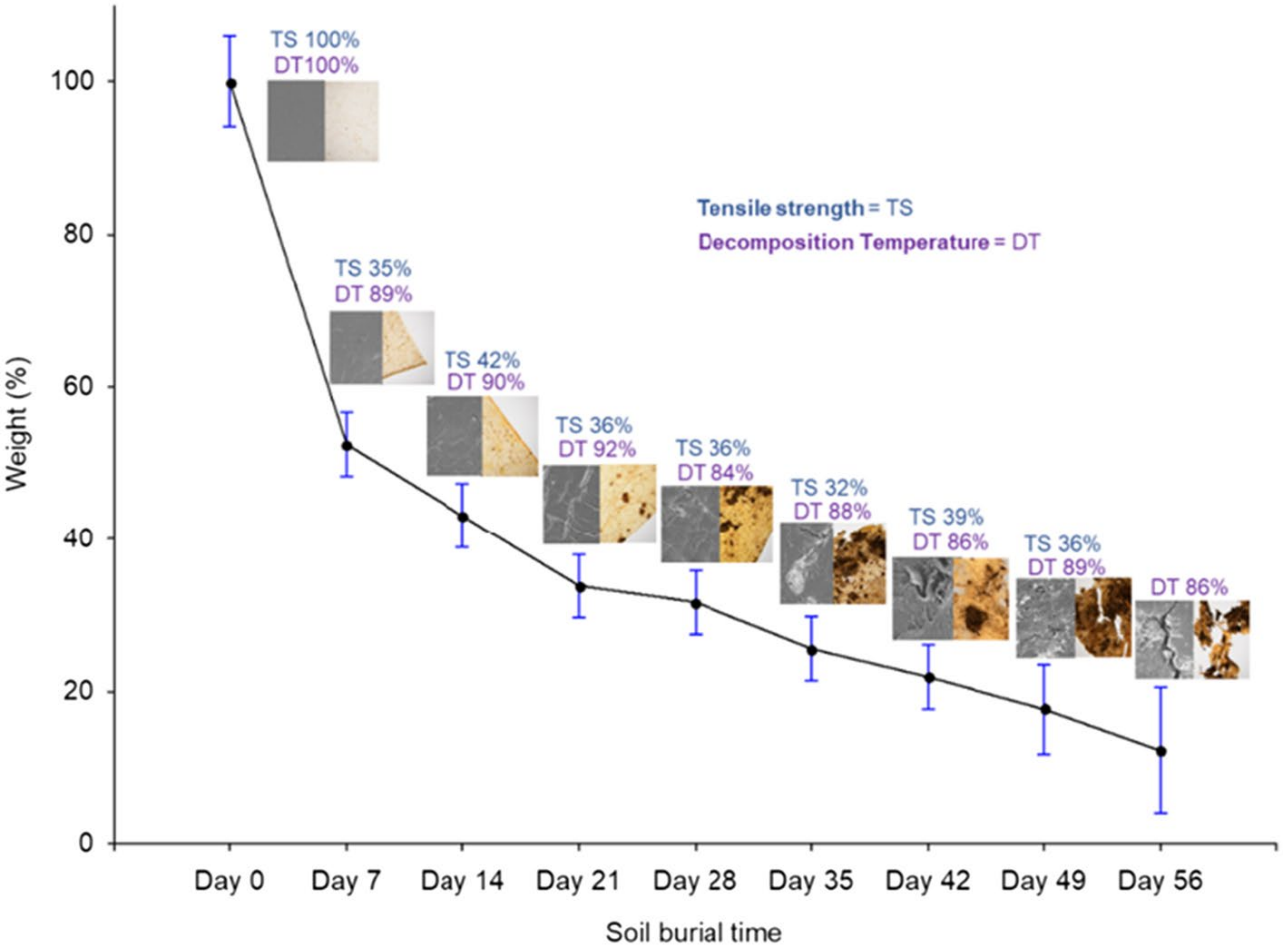
Changes in the different aspects of cellulose films in relation to changes in weight of cellulose films with the increase of soil burial time (First experiment).

The inference that could be derived from all the findings was that every instance was related to one another (Fig. 9). As the burial time increased, there was a gradual weight loss in the cellulose films. This was accompanied by important changes in the visual aspect; a concurrent rise in surface roughness, which led to the formation of cracks and holes and increased susceptibility to further sample disintegration (Visual, SEM, and Stereo observations). The leading causes were the loss of glycerol from the films (FTIR and TGA results) and the spread of microorganisms on the surface (FTIR and Stereo results). It coincided with a notable change in the mechanical and thermal characteristics of the films.

## Conclusions

Given their convenience and broad spectrum of applications, plastics have become an essential part of our lives. Bioplastics are one of the most promising solutions to reduce the unintended health and environmental consequences of the excessive use of plastics and permanent litter. In this study, we prepared bioplastic films from low-quality cotton fibers and investigated the degradation of these films in a moisture-controlled soil environment. During the biodegradation process, cellulose films underwent significant changes in physicochemical, thermal, and mechanical properties. They were completely degraded in 63 days and 112 days in late summer and spring weather conditions, respectively. Over time, the smooth surface of cellulose films became rougher, irregular, and uneven with holes and fractures, and intense changes in surface morphology were observed in the later stages of the experiments. Microscopic observations confirmed that microbial growth on cellulose films was favored by the hydrophilicity and amorphous nature of regenerated cellulose films. The FTIR results also suggested the growth of microorganisms in cellulose films, the removal of glycerol, and the adhesion of soil particles. The degradation of cellulose films was also associated with a significant reduction in thermal stability and weakening of cellulose films. The initial changes in tensile properties were related to the removal of glycerol, while the later changes were associated with the microbial assimilation of cellulose. It was also noticed that the degradation rate depended on the soil temperature and temperature fluctuations in the environment, which impacted microbial activities and survival. All these results suggest that cellulose films naturally degrade when disposed of in terrestrial environments, and therefore, after cellulose films are used, landfill disposal would be suitable. It demonstrated the fast-closed carbon cycle of cellulose films. In addition, the potential of using cellulose films as agricultural mulches for sustainable agriculture techniques is demonstrated in our current study. A key concern for growers is the decomposition of biodegradable mulch film following the growing season, as a slow breakdown can lead to residue buildup, which can hinder field operations or cause an imbalanced supply of nutrients in subsequent growing seasons^40^. Biodegradable mulches have been reported to decompose within 1–2 years after being introduced into the soil^41,42^. Furthermore, the majority of biodegradable mulch products have an issue with petroleum-derived polymers they contain, which prevents them from being used in USA certified organic cultivation^43^. Consequently, it would be beneficial to develop entirely biobased mulch films that have fast biodegradation capabilities. Therefore, we are interested in exploring potential utilization of cellulose film as soil cover mulch. We have attempted to investigate the functionality of the films as agricultural mulches and their subsequent incorporation in the soil for degradation at the end of the crop season. This study is currently in progress.

## Material and methods

### Materials

N,N-dimethylacetamide (DMAc) (99 + % extra pure, A0403006) and anhydrous lithium chloride (LiCl 99  %, A0386841) were purchased from Acros Organics™ (NJ, USA). Glycerol (certified ACS, 202397) was purchased from Fisher Scientific™ (MA, USA). Low-quality cotton was obtained from the Fiber and Biopolymer Research Institute, Texas Tech University (Lubbock, TX, USA). Organic potting soil (All-Natural Premium Outdoor Potting Mix, Kellogg Garden Products Corporate, CA, USA) was purchased from Home Depot. It contained recycled forest products, perlite, sand, ground dolomitic limestone, bat guano, kelp meal, peat moss, coir, poultry manure, and plant nutrients: total nitrogen 0.3%, available phosphate 0.1%, soluble potash 0.1%. Deionized (DI) water was purchased from AquaOne (Amarillo, TX, USA).

### Methods

#### Film preparation

Films were prepared following the dissolution, gelation, regeneration, plasticization, and hot-pressing protocol reported in our previous study^14^. In summary, scoured and bleached low-quality cotton fibers were dissolved in a DMAc/LiCl solvent system, and 110 ml of the viscous solution was cast in glass molds. Upon gelation at room temperature, the resultant flexible, transparent, and sheet-like gel was regenerated in water, plasticized with 30% aqueous glycerol solution, and subsequently hot-pressed. Before the soil burial experiments, the cellulose films were conditioned under ambient room conditions (relative humidity of 30 ± 2% and temperature of 21 ± 1 °C) for at least 48 h, and the length, width, and weight of individual cellulose films were recorded.

#### Degradation of films during soil burial

The degradation study was conducted in Lubbock, Texas, which is located at 33.5846° N and 101.8456° W. Lubbock experiences hot summers and chilly winters due to its semi-arid environment. The degradation behavior of cellulose films was studied by following the methods reported by Dharmalingam et al. and Jayaramudu et al. with slight modifications^15,44^*.* Plastic trays (length × width × height approximately 45 × 35 × 15 cm) were selected, and drainage holes were made in the bottom. Then, soil beds of 10 cm depth were prepared by adding premoistened organic potting soil. The soil beds were conditioned for 7 days at a 12 ± 2% moisture level, and water was added as needed to maintain the soil moisture level. Cellulose films were buried in the moist soil beds 5 cm below the soil surface, and the soil was gently hand-compacted to achieve good contact between samples and soil. Then, soil trays were placed inside a high tunnel used for growing vegetables. The soil moisture content of each tray was recorded daily using a digital soil moisture meter (DSMM500, General Tools & Instruments, USA), and water was added using a watering can as needed (approximately 500 ml every two days) to maintain 12 ± 2% of the soil moisture level Two degradation experiments were conducted in late Summer (11th August to 13th October) and Spring weather (24th March to 24th July) conditions with two individual replicates. Each tray/replicate contained 20 samples, and two cellulose films were retrieved from each tray at each retrieval.

#### Sample characterization

After each retrieval, the cellulose films were gently cleaned using a brush to remove attached soil particles, and visual images were collected. Then, the cleaned films were conditioned at ambient room conditions for at least 48 h, and weight measurements were recorded. Before further characterization, the films were conditioned for at least 48 h in an environment of 65 ± 2% relative humidity and 21 ± 1 °C.

*Weight change during sample degradation:* The percent weight change in each sample due to soil burial-induced degradation was calculated using the following equation:

$$Weight\, loss (\mathrm{\%})=\frac{Initial\, weight -Weight\, after\, retrieval}{Initial weight}\times 100$$

*Surface morphology:* Degradation-associated changes in cellulose films were observed using stereomicroscope (SMZ800N, Nikon Corporation, Tokyo, Japan), scanning electron microscope (SEM, TM‐1000, Hitachi, Japan), and field emission scanning electron microscope (FESEM, S/N 4300, Hitachi, Chiyoda, Tokyo, Japan). For stereomicroscopy, the samples were directly placed on the stage and visualized with a 1X objective lens. For SEM, the samples were mounted on the sample stage covered with carbon tape (Ted Pella Inc., Redding, CA, USA) and visualized at 500 magnifications with an accelerating voltage of 15 kV. For FESEM, samples were coated with iridium, and images were recorded with a 5 kV accelerating voltage at 3.5 K and 5 K magnifications. Quartz PCI Imaging software (Version 8, Quartz Imaging Corp., Vancouver, Canada) (https://www.quartzimaging.com/pci-microscope-imaging-software.html) was used to analyze the recorded images.

*Thermogravimetric Analysis (TGA):* The thermal properties of the cellulose films were analyzed using a Pyris 1 thermogravimetric analyzer (PerkinElmer, MA, USA) equipped with an autosampler. Approximately 10 mg of each sample was placed in the crucible of the autosampler, and thermograms were recorded between 37 and 600 °C with a heating rate of 10 °C/min under a nitrogen flow of 20 ml/min. The percent weight loss, first derivatives of the thermograms (DTG), and peak decomposition temperature for each sample were calculated using Pyris data analysis software (PerkinElmer, MA, USA).

*Fourier Transform Infrared Spectroscopy *(*FTIR spectroscopy*)*:* FTIR spectra of cellulose films were recorded using an FTIR spectrometer (Spectrum 400, PerkinElmer, MA, USA) equipped with a ZnSe diamond crystal and a pressure arm. Spectral data between 4000 and 650 cm^−1^ were recorded at 4 cm^−1^ spectral resolution with 32 coadded scans. Spectra collected from cellulose films were baseline corrected and normalized using Spectrum software (PerkinElmer, MA, USA) and subsequently compared to investigate degradation-associated changes.

*Tensile Testing:* The tensile properties of cellulose films were recorded according to ASTM D638-14 using the Multi-Test 2.5-dV(u) Test System (Mecmesin, UK) at a gripping distance of 65 mm and speed of 50 ± 10 mm/min. We were able to prepare two tensile specimens usually from each cellulose film. Nevertheless, it was not feasible to prepare two tensile specimens at the later stage of degradation from each cellulose film, as the cellulose films were deteriorating. Depending on the integrity and shape of the samples, one or two specimens were prepared from each sample by an ASTM D638- Type IV tensile die and a manual clicker press (Qualitest, USA: https://www.worldoftest.com/). Overall, we were able to prepare a total of 6–8 tensile specimens for each retrieval.

*Statistical analysis:* One‐way analysis of variance (ANOVA) was conducted with sample retrieval time as a factor using STATISTICA (Version 13.3; March 2021; TIBCO Software, CA, USA) (https://docs.tibco.com/products/tibco-statistica-13-3-0) to determine the significant difference at 95% confidence intervals (CIs).

### Supplementary Information

Supplementary Information.

## Data Availability

All relevant data are included in the manuscript. Data can be available from the corresponding author upon request.
