# Supplementary material for: Soil burial-induced degradation of cellulose films in a moisture-controlled environment
Source: Sci Rep. 2024 Mar 22;14:6921. doi: 10.1038/s41598-024-57436-w (PMC10960015; doi:10.1038/s41598-024-57436-w)
Supplement: Supplementary file 1 — Supplementary Information. [file 41598_2024_57436_MOESM1_ESM.docx]

Soil burial-induced degradation of cellulose films in a moisture-controlled environment

**Shaida S. Rumi, Sumedha Liyanage, Noureddine Abidi***

Fiber and Biopolymer Research Institute, Department of Plant and Soil Science, Texas Tech University, Lubbock, Texas, USA 79409

*Corresponding author: Noureddine Abidi (E-mail: [noureddine.abidi@ttu.edu](mailto:noureddine.abidi@ttu.edu))

ORCID: Sumedha Liyanage ([0000-0002-8980-7817](https://na01.safelinks.protection.outlook.com/?url=http%3A%2F%2Forcid.org%2F0000-0002-8980-7817&data=02%7C01%7Csumedha.liyanage%40ttu.edu%7C0f122133eb6e4349471f08d520bb37cc%7C178a51bf8b2049ffb65556245d5c173c%7C0%7C0%7C636450910819843610&sdata=d%2BrpU2b9MbQGRXjfBQ5t7Ur%2BwMSz2xK2iAoD0mXuW%2BQ%3D&reserved=0)); Noureddine Abidi ([0000-0001-5642-6332](http://orcid.org/0000-0001-5642-6332))

**Supplementary Material**

**Figure S1**

| 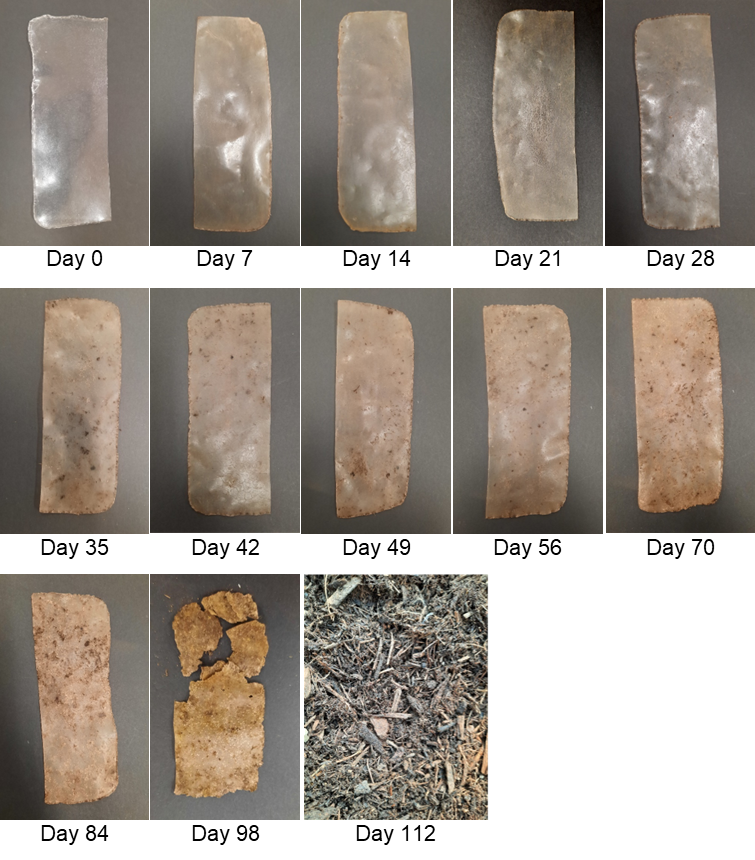 |
| --- |

**Figure S1.** Visual changes of soil-buried cellulose films retrieved during the second experiment (control film (day 0) and films retrieved on days 7, 14, 21, 28, 35, 42, 49, 56, 70, 84, 98, and 112).

**Figure S2**

| 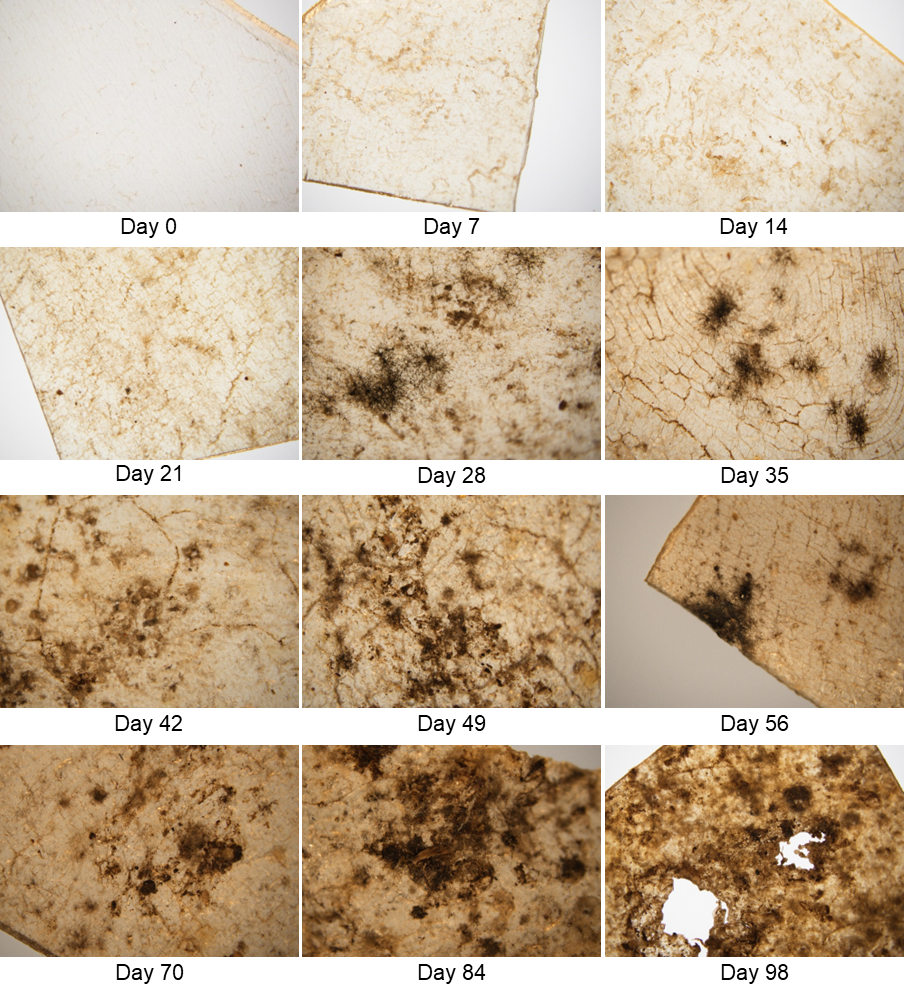 |
| --- |

**Figure S2.** Stereomicroscopy images of cellulose films retrieved during the second soil burial experiment.

**Figure S3**

| **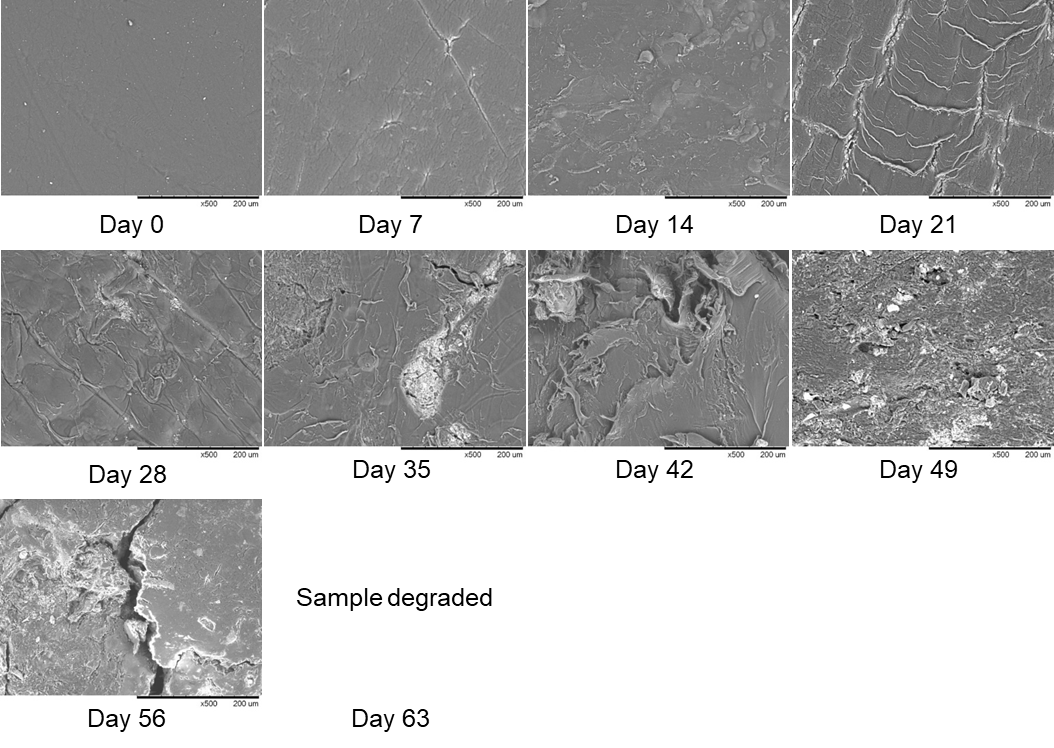** |
| --- |

**Figure S3.** Scanning electron microscopy (SEM) analysis of retrieved cellulose films from the first soil burial experiment. SEM micrographs of control and retrieved cellulose films on days 7, 14, 21, 28, 35, 42, 49, and 56.

**Figure S4**

| 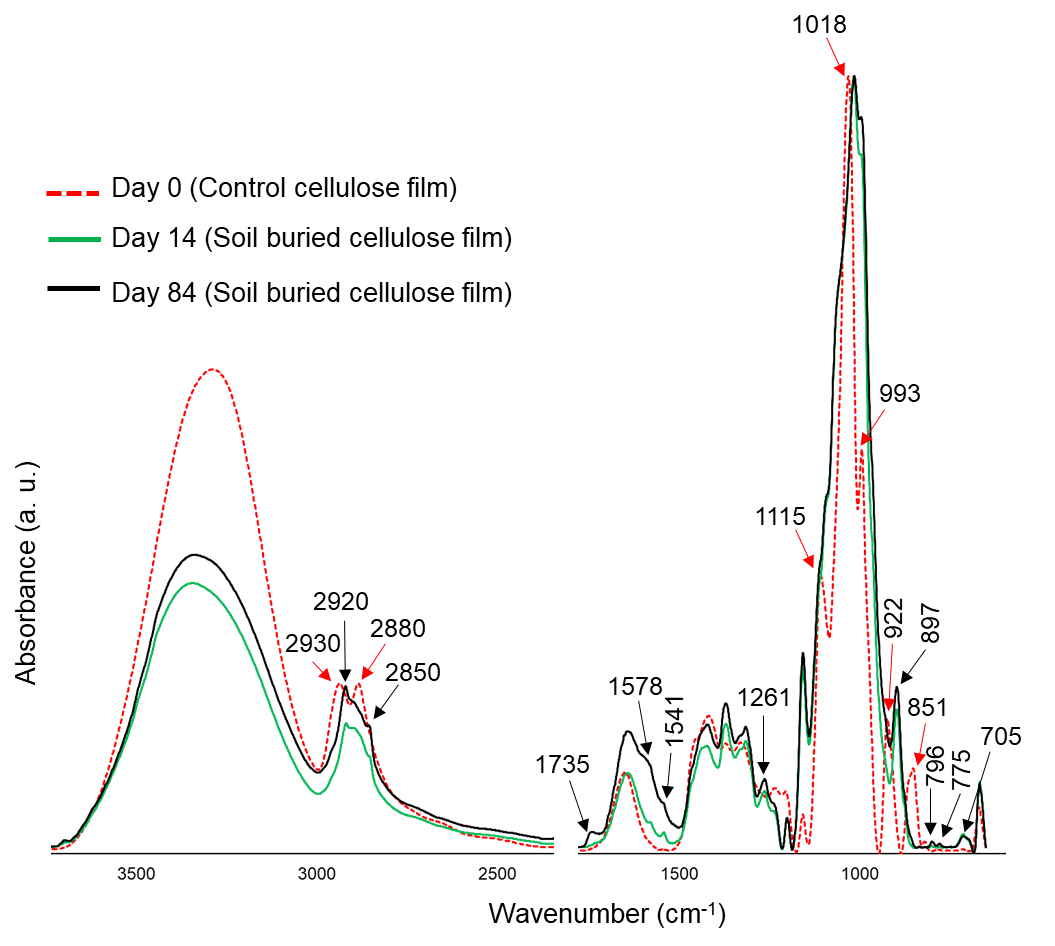 |
| --- |

**Figure S4.** FTIR spectra acquired from control cellulose film (day-0) and the films retrieved from the second soil burial experiment at days 7 and 84 representing early and late degradation stages, respectively.

**Figure S5**

| **a.** | 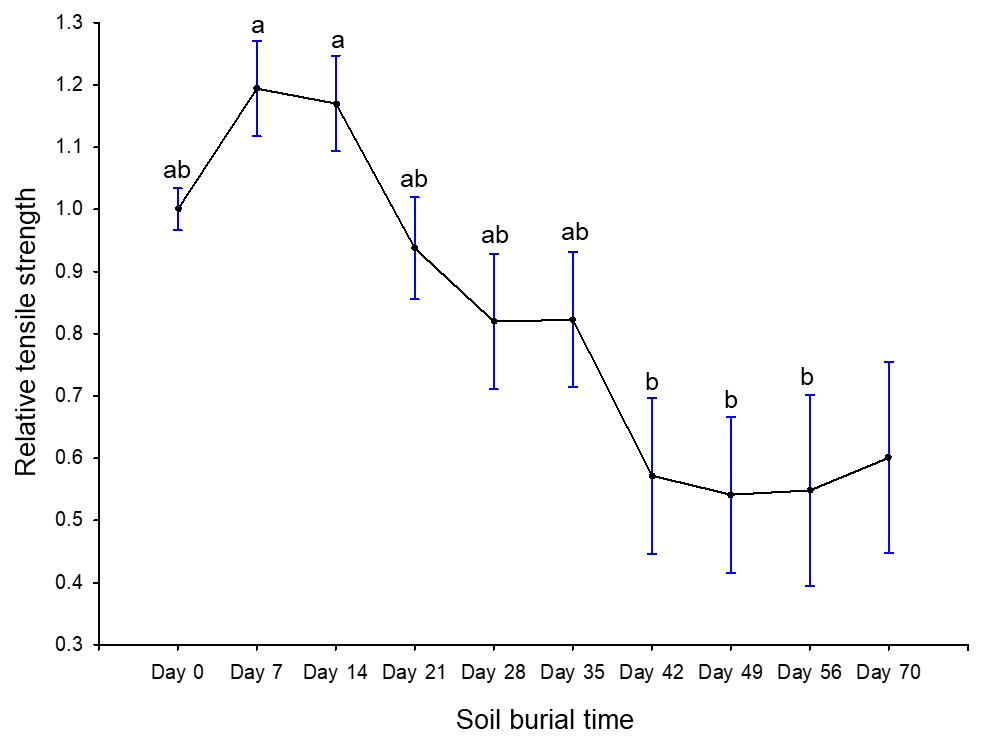 |
| --- | --- |
| **b.** | 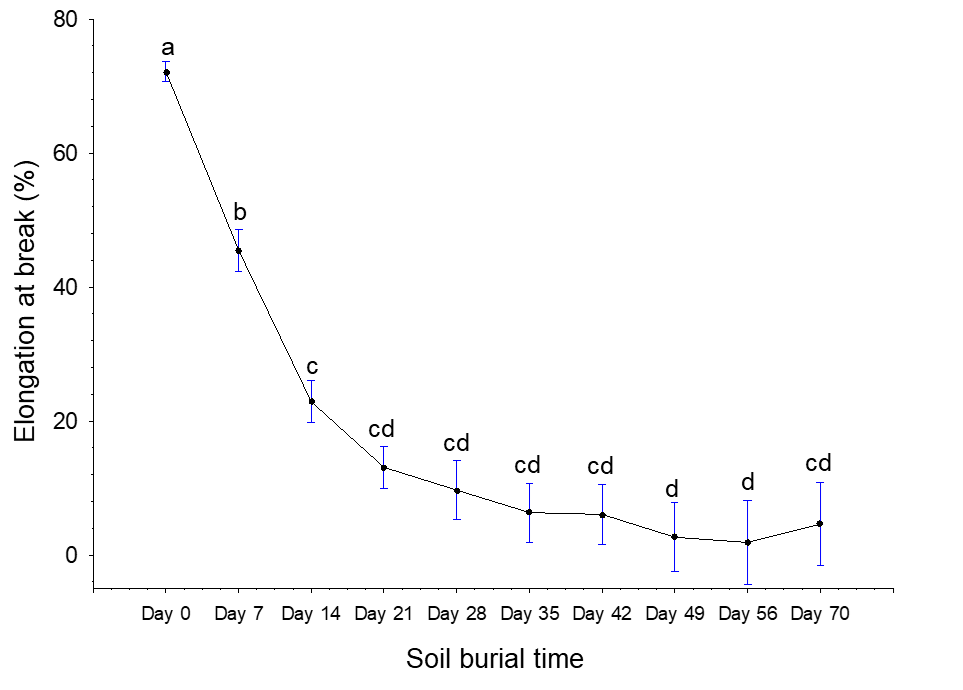 |
| **c.** | 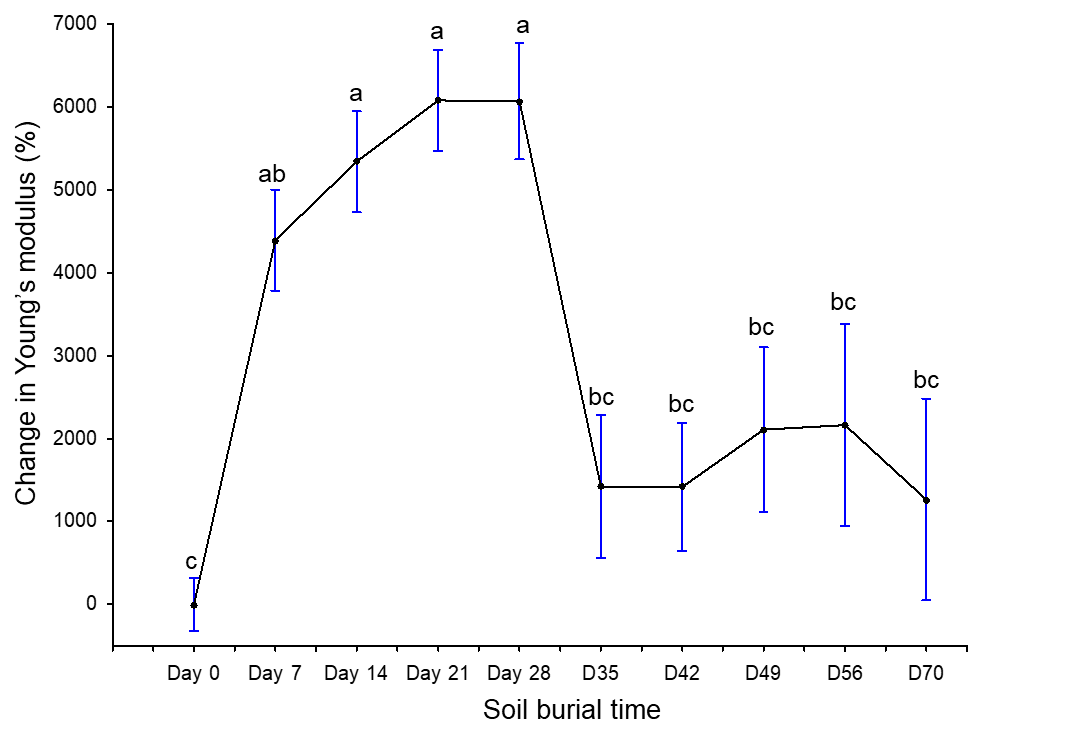 |

**Figure S5.** The changes in tensile properties of cellulose films retrieved during the second soil burial degradation study. (a) Relative tensile strength, (b) elongation at break (%), and (c) change in Young’s modulus. Values not followed by the same letter significantly differ at α = 0.05.

**Figure S6**

| **a.** | 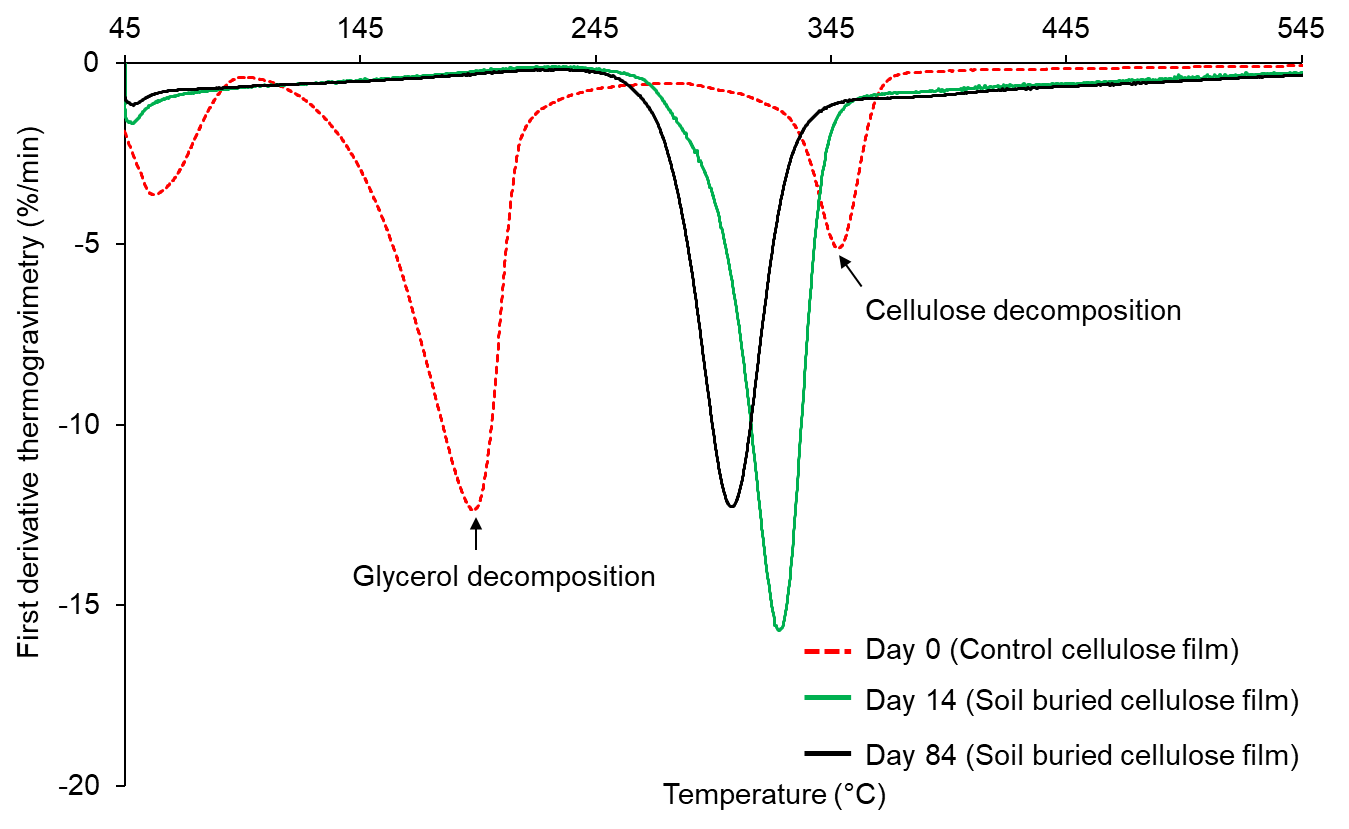 |
| --- | --- |
| **b.** | 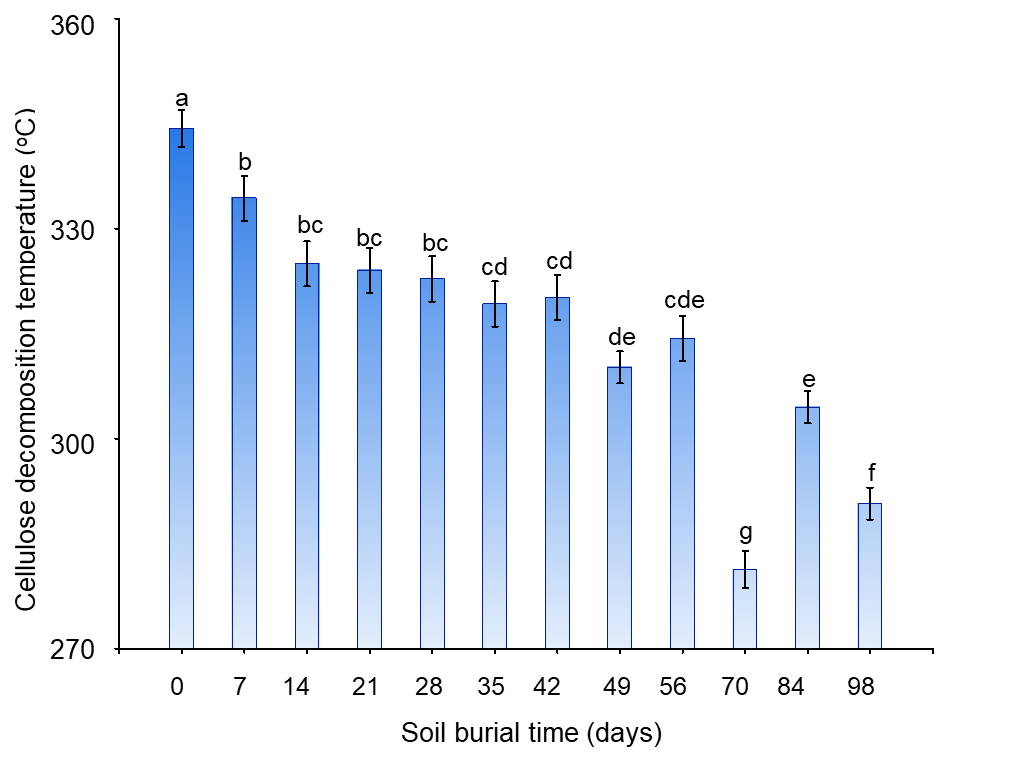 |

**Figure S6.** Thermogravimetric analysis (TGA) of the cellulose films retrieved during the second soil burial experiment. (a) First derivative thermogravimetry of the control and the soil buried cellulose films retrieved at days 7 and 84. (b) Changes in cellulose decomposition temperatures of the control and retrieved soil buried cellulose films. Values not followed by the same letter are significantly different at α = 0.05.
